# Innate Lymphoid Cells in Skin Homeostasis and Malignancy

**DOI:** 10.3389/fimmu.2021.758522

**Published:** 2021-10-08

**Authors:** Marek Wagner, Shigeo Koyasu

**Affiliations:** ^1^ Laboratory for Immune Cell Systems, RIKEN Center for Integrative Medical Sciences, Yokohama, Japan; ^2^ Department of Biomedicine, University of Bergen, Bergen, Norway

**Keywords:** innate lymphoid cells, skin, skin cancer, melanoma, immunity, immunosurveillance

## Abstract

Innate lymphoid cells (ILCs) are mostly tissue resident lymphocytes that are preferentially enriched in barrier tissues such as the skin. Although they lack the expression of somatically rearranged antigen receptors present on T and B cells, ILCs partake in multiple immune pathways by regulating tissue inflammation and potentiating adaptive immunity. Emerging evidence indicates that ILCs play a critical role in the control of melanoma, a type of skin malignancy thought to trigger immunity mediated mainly by adaptive immune responses. Here, we compile our current understanding of ILCs with regard to their role as the first line of defence against melanoma development and progression. We also discuss areas that merit further investigation. We envisage that the possibility to harness therapeutic potential of ILCs might benefit patients suffering from skin malignancies such as melanoma.

## Introduction

The family of innate lymphoid cells (ILCs) comprises a heterogeneous population of immune cells harboring pleiotropic functions. Based on the expression of signature cytokines and an assembly of transcription factors, they have been divided into five subsets, namely natural killer (NK) cells, group 1 ILCs (ILC1s), ILC2s, ILC3s and lymphoid tissue inducer (LTi) cells (1). Accordingly, ILC1s secrete type 1 cytokines such as IFN-γ and TNF-α. They require the expression of T-bet, a T-box transcription factor, for development and function, but unlike NK cells are not cytotoxic and can develop in the absence of eomesodermin (Eomes), another T-box transcription factor that is homologous to T-bet and essential for NK cell differentiation (1). ILC2s secrete type 2 cytokines, including IL-4, IL-5 and IL-13 and depend on the expression of GATA3 and RORα (2, 3). Not least of all, ILC3s and LTi cells produce IL-22 and/or IL-17 and require RORγt. LTi cells, however, also produce lymphotoxin (LT), a member of the TNF family of cytokines and arise from a different developmental pathway than ILC3s (1). It should be noted, however, that in the human peripheral blood ILC3s are immature and rather represented by a population of ILC progenitors (ILCPs) (4).

The heterogeneity and diversity of ILCs might further increase owing to their plastic potential (4–6). For example, the combination of IL-1β and IL-12 has been found to induce the transdifferentiation of human ILC2s into IFN-γ-producing cells resembling ILC1s (7). It has also been demonstrated that IL-4 can reverse that phenotype converting cells reminiscent of human ILC1s back to ILC2s (8). Recent study has also reported the transdifferentiation of human cutaneous ILC2s into IL-17-producing cells resembling ILC3 (9). The plastic potential of ILCs might therefore serve as an important feature of their ability to adapt rapidly to the fluctuating levels of environmental stimuli. A growing body of evidence indicates that environmental stimuli ingrain the phenotype of ILCs and thus their function (10, 11). With that in mind, ILCs have been equipped with receptors to sample the environment and react against threats to tissue integrity through the production of cytokines and chemokines. They are promptly activated by stress signals and various epithelial- and myeloid cell-derived cytokines, rather than by antigens as T and B cells (12, 13).

Similarly to NK cells, ILC1s require IL-15 for their development. Additionally, they are both activated by IL-12, IL-18 and IL-15 (14, 15). Whereas IL-12 and IL-18 are secreted by monocytes and activated DCs, IL-15 is produced by activated monocytes and macrophages as well as a variety of non-hematopoietic cells, including but not limited to epithelial and fibroblast cell lines (15). ILC2s, on the other hand, respond primarily to IL-33, IL-25 and thymic stromal lymphopoietin (TSLP, combined with IL-33), which are produced by numerous cell types (2, 3, 15, 16). For example, expression of IL-33 can be found in epithelial and endothelial cells, smooth muscle cells, fibroblasts, macrophages and activated DCs (15). Expression of TSLP, however, typifies epithelial cells in the barrier tissues such as the skin, whereas activated Th2 cells together with macrophages, mast cells, eosinophils, basophils and fibroblasts as well as skin epithelial cells, tuft cells, and endothelial cells produce IL-25 (15). Last in order, ILC3s and LTi cells are activated by IL-1β and IL-23 produced by activated DCs and macrophages (17, 18).

In contrast to NK cells, which circulate in the body and are particularly detected in the peripheral blood, the remaining ILC subsets are mostly tissue resident and preferentially enriched in barrier tissues such as the skin. The involvement of NK cells in antitumor immunity is unquestionable (19–22). Their abundance in the circulation correlates with decreased metastatic potential in numerous human cancers (23, 24). However, our understanding of the role and function of the remaining ILC subsets in skin malignancies is still in its infancy. The most aggressive form of skin cancer, melanoma, originates in melanocytes, which are found in the skin, eyes and hair. Although less common than squamous and basal cell carcinoma, melanoma, if left untreated at an early stage, is far more perilous because of its ability to spread more rapidly to distant organs. Melanoma has been thought to trigger immunity mediated mainly by adaptive immune responses. To what extent innate immunity, and in particular, innate lymphoid cells impact melanoma is not well understood. Here, we summarize recent insights into the unique features and functions of ILCs pertaining to their role in the protection from melanoma development and progression. We also discuss areas that require further investigation and highlight discoveries, which could have implications for the development of new therapeutic strategies.

## ILCs in the Skin

The skin is the largest organ of the body (25). It provides thermal insulation and physical protection from injury and infection. It also stores water, stacks the majority of the body fat and produces vitamin D. The skin is composed of three anatomically distinct layers: epidermis, dermis, and subcutis. The outermost layer of the skin, epidermis, is composed of squamous and basal cell keratinocytes, melanocytes as well as Merkel’s cells, which serve as mechanoreceptors. Keratinocytes, which are the most abundant cells within the epidermis, produce the key structural material, keratin, as well as lipids responsible for the formation of the epidermal water barrier. They are also responsible for converting 7-dehydrocholesterol to vitamin D with the assistance of ultraviolet B (UVB) light from the sun. UVB light also stimulates melanocytes to secrete melanin, which is responsible for the pigment of the skin (26). The middle and by far the thickest layer of the skin, dermis (together with epidermis called cutis), is primarily composed of fibroblasts but also contains blood and lymphatic vessels, nerves as well as epidermally derived appendages including hair follicles, sudoriferous (or sweat) glands and sebaceous glands, which are deeply integrated into the fabric of connective tissue. Connected to epidermis and probably the most understudied is the deepest layer of the skin, subcutis (sometimes referred to as hypodermis), made of fat and connective tissue, which also contains extensive vasculature. However, it should be noted that mouse and human skin are quite distinct in terms of structure (27). Whereas, mouse epidermis is usually composed of only 3 cell layers (< 25μm in thickness), human epidermis is often formed of 6–10 cell layers (> 50 μm in thickness) (27). Similarly, human dermis is much thicker than mouse dermis. On the other hand, although thinner, mouse skin has more densely distributed hair follicles and contains a cutaneous muscle layer called, panniculus carnosus (27).

The skin also represents a highly specialized immunological niche with immune cells closely interacting with non-hematopoietic parenchymal cells to ensure the maintenance of the barrier function (25). For example, in an event of an insult, non-hematopoietic parenchymal cells regulate recruitment, activation and tissue residency of immune cells, whereas immune cells secrete cytokines as well as growth factors necessary for the prevention of infection and tissue reconstruction. Traversed by blood and lymphatic vasculature, dermis contains most of the immune cells in the skin, including several subsets of dermal DCs, CD4^+^ and CD8^+^ T cells, γδT cells, B cells, macrophages, basophils, eosinophils, mast cells and NK cells (25). Accumulated evidence has demonstrated that ILCs, which are preferentially enriched in the skin, play an important role in barrier tissue immunity (28). Beside NK cells, ILC2s were the first ILCs to be discovered in the skin, both in mice and humans (29). Although ILCs can be detected in epidermis and dermis, the majority were identified in the deeper layer of the skin with ILC2s comprising 5-10% of all CD45^+^ cells in mice (30). Recently, transcriptome analysis of bulk and single-cell RNA-sequencing data demonstrated enrichment of ILCs expressing genes associated with ILC2s (e.g. *Gata3* and *Il5*) in subcutis, whereas ILCs in epidermis were found to predominantly express genes associated with ILC3s/LTi cells (e.g. *Rorc* and *Lta*) but also ILC2s (e.g. *Il13* and *Il2*). On the other hand, ILCs in dermis shared resemblance with ILCs from epidermis and subcutis (31).

Tissue residency of ILCs in the skin might be governed by the tissue-derived cytokines. For example, *Il7^-/-^* mice have been found devoid of ILC2s in subcutis, whereas the number of ILCs in epidermis and dermis was moderately reduced when compared with wild-type mice. However, complete loss of ILCs has been observed in the skin of *Il7^-/-^Tslp^-/-^* mice indicating collaborative regulation of skin residency by IL-7 and TSLP (31). The pattern of chemokine receptors expressed by ILCs might also be involved in the regulation of tissue residency. For example, ILCs that reside in the mouse epidermis express CCR6, which serves as a receptor for CCL20 that is highly expressed in the upper portion of the hair follicles (32). Hair follicles have therefore been portrayed as epicenters that recruit and position ILCs in the skin. It has also been demonstrated that hair follicles provide cytokines such as IL-7 and TSLP, which ILCs depend on for maintenance (33). Furthermore, ILCs present in the skin-draining lymph nodes that express CCR10 have been found to migrate to the skin in a CCR10-dependent manner (34).

From another angle, using mice expressing eGFP under the control of the locus of CXCR6, an analysis of the potential immunosurveillance activity in the skin revealed that ILC2s patrol their environment with an average speed similar to that of dermal DCs (i.e. 5 μm/min) (30). An analysis of the interactions with other cell types demonstrated that ILC2s strongly interact with mast cells and suppress IgE-dependent cytokine production by mast cells through the release of IL-13 (30). However, human ILC2s have also been found to induce strong proinflammatory responses following stimulation with prostaglandin D2 (PGD2) produced by mast cells. Indeed, activation of human ILC2s by PGD2 has increased their migration and upregulated the expression of IL-33 and IL-25 receptor subunits (ST2 and 17A, respectively) as well as induced production of type 2 and other cytokines such as IL-3, IL-8, IL-9, IL-21, GM-CSF and CSF-1 (18). Of note, mast cells are likely not the only source of PGD2. Although studied not specifically in the skin, ILC2s and epithelial cells have also been found to produce PGD2 (35–37).

The involvement of ILC1s and ILC3s in skin homeostasis requires further investigation. It should be noted, however, that ILC3s might play a critical role in the maintenance of tolerance towards skin microbiota (38). Nevertheless, they have also been associated with the development of psoriasis, an immune-mediated chronic disorder of the skin (39–41).

## ILCs and Skin Wound Healing

The skin has evolved precise and orderly mechanisms to close breaches to its integrity in a process known as the wound healing response. Human ILC2s isolated from the skin have been typified by an increased gene expression of amphiregulin when compared with those purified from blood. Since amphiregulin aids tissue repair, it has been suggested that ILC2s in the skin are involved in wound healing response (42). This notion has recently been supported by a study, which found that ILC2s are important in the reepithelialization of cutaneous wounds. Indeed, elevated numbers of ILC2s have been found at the site of injury five days after wound induction. Importantly, impaired reepithelialization accompanied by diminished numbers of activated ILC2s has been observed in IL-33-deficient mice at the site of injury when compared with wild-type mice. However, treatment with recombinant IL-33 has significantly increased reepithelialization five days after wound induction (43).

Additionally, presence of IL-17A, which is produced among others by ILC3s, has also been found in human wounds. Interestingly, delayed wound closure has been observed in *Il17a^-/-^* mice (44). Although dendritic epidermal T cells (DETC) have been portrayed as an important source of IL-17A in the study, it is possible that ILC3s might also be engaged in the wound healing response, since impeded wound closure has been more pronounced in *Il17a^-/-^* mice when compared with *Tcrd^-/-^* mice, which lack DETC (44). Indeed, it has recently been revealed that damage to the skin activates Notch signaling, which in turn, induces recruitment of RORγ^+^ ILC3s through the production of TNF-α. Additionally, RORγ^+^ ILC3s have been found to produce IL-17F and CCL3 (also known as MIP1α) involved in the healing response through the regulation of epidermal proliferation and macrophage recruitment into dermis (45).

More than three decades ago, Dvorak suggested that cellular and biochemical processes associated with wound healing, although lost at the level of regulation, are reminiscent of the tumor stroma development. He thus coined the phrase that tumors are “wounds that do not heal” (46). Although ILCs seem to play an active role in wound healing, the nature of their responses in tumors has only recently begun to be unveiled.

## ILCs in Melanoma

### NK Cells and ILC1s

Among all ILCs, NK cells are certainly the most extensively studied mediators of immune responses against cancer (23, 47). Responses against melanoma in particular have also been detailed [Tarazona et al. for comprehensive review (19)]. Briefly, numerous studies have demonstrated that NK cells are able to distinguish and destroy melanoma cells *in vitro* (19, 48). The tumor suppressive role of NK cells has also been demonstrated using variety of *in vivo* mouse models (19). Last but not least, evaluation of NK cell alterations in melanoma patients such as down-regulation of activating receptors and exhaustion of NK cells has indicated establishment of escape mechanisms by melanoma cells to evade NK cell-mediated recognition and destruction (19, 20). Interestingly, the abundance of CD56^bright^ NK cells in the peripheral blood obtained from late stage (III/IV) melanoma patients has recently been found to negatively correlate with overall patient survival (49).

The studies of NK cells have generally stressed their cytotoxic role mediated by the release of cytotoxic granules containing perforin (PFN) and granzyme B (GrB) or by the engagement with death receptors that initiate caspase cascade. Portrayed as the first line of defence, NK cells have been viewed as innate immune cells that operate at an earlier stage than T and B cells. However, involvement of NK cells in T cell-mediated immunity has also been demonstrated (50, 51). Recently, NK cells have been found to attract XCR1^+^ DCs that are critical for T cell–mediated immunity to melanoma tumors through the secretion of XCL1, XCL2 and CCL5 (51). NK cell frequency has also been found to correlate with cross-presenting DCs in melanoma tumors as well as with responsiveness to anti-PD-1 immunotherapy in patients and their increased overall survival (50). A broader role for NK cells, beyond their direct cytotoxic function, has therefore been proposed.

In contrast to NK cells, little is known about the role of ILC1s in melanoma. Recently, enrichment of ILC1s, although with impaired IFN-γ production capabilities, has been observed in both peripheral blood and tumor cell-infiltrated lymph nodes from melanoma patients (52). Interestingly, IFN-γ signaling in cancer and immune cells has been found to oppose each other, in order to develop a regulatory relationship that restrains both innate and adaptive immune responses. Inhibition of tumor IFN-γ signaling has been found to decrease IFN-stimulated genes (ISGs) in cancer cells and increase ISGs in immune cells by enhancing IFN-γ production by exhausted T cells. In tumors with neoantigens or MHC-I loss, including melanoma, exhausted T cells utilize IFN-γ to stimulate maturation of innate immune cells, more specifically, a population of PD1^+^TRAIL^+^ ILC1s (53). The possibility to inhibit tumor IFN-γ signaling and, at the same time, disable an inhibitory circuit impacting PD1 and TRAIL has been suggested to promote innate immune killing.

### ILC2s

Although the number of studies focusing on the role of ILC2s in tumor immunity has increased, many aspects related to the mechanisms behind their antitumor function still remain to be clarified.

ILC2s have been associated with the induction of apoptosis mediated through CXCR2 signaling in melanoma tumors engineered to express IL-33 (54). Furthermore, ILC2s are known to produce IL-5, which is essential for the expansion of eosinophils, since its localized production stimulates tissue eosinophilia (20, 55). In mice, ILC2s have been found to maintain sufficient numbers of eosinophils in the lungs through the production of IL-5 in response to melanoma invasion. Additionally, genetic blockade or antibody-mediated neutralization of IL-5 has been shown to impair eosinophil recruitment into the lungs leading to an increased metastatic dissemination of melanoma cells (56). Recently, ILC2-derived granulocyte macrophage-colony stimulating factor (GM-CSF) has been shown to contribute to the recruitment and activation of eosinophils into melanoma tumors (57). Since ILC2s have been found to express PD-1, the combination of anti-PD-1 blocking antibodies together with IL-33 improved anti-tumor responses through the expansion of tumor-infiltrating ILC2s accompanied by eosinophils. Importantly, deletion of NK cells, ILC1 and/or ILC3s had no impact on either tumor growth or survival of the mice, suggesting that ILC2s play the key role in restricting the development of melanoma tumors (57).

The exact role of eosinophils in melanoma remains to be determined, however, increased tumor growth and metastatic potential have been demonstrated following an antibody-mediated depletion of eosinophils in IL-33-treated mice bearing melanoma tumors (58). The cytotoxic activity of eosinophils has been attributed to the secretory granules made of major basic protein 1 (MBP-1) and MBP-2, eosinophil cationic protein, eosin-derived neurotoxin, and eosinophil peroxidase (55). Indeed, MBP^+^ eosinophils have been found to clear metastatic melanoma cells in the mouse lungs, whereas the lysates of MBP^+^ eosinophils have turned out cytotoxic *in vitro* when co-cultured with cancer cells (59). Importantly, we have observed a significant correlation between an overall survival and the expression of IL-33 as well as an eosinophil marker SIGLEC8 in patients suffering from melanoma. It should be noted, however, that the expression of IL-33 and SIGLEC8 has been found to demonstrate different survival prognosis in diverse types of cancer, with better survival outcomes in melanoma patients but not in those with pancreatic adenocarcinoma and lung squamous cell carcinoma (60). Increased median overall survival has also been shown in patients with metastatic melanoma presenting a high number of eosinophils in the circulation during immune checkpoint blockade therapy (ICB). Therefore, eosinophilia has been suggested to serve as a potential prognostic marker for melanoma patients during ICB (61, 62).

A growing body of evidence indicates that immune cells can also be influenced by the metabolism of cancer cells, and the cellular and molecular mechanisms are only now becoming determined (20). We have also found that the production of lactic acid by melanoma cells greatly impairs eosinophil-mediated antitumor response regulated by ILC2s. B16F10 melanoma tumors with diminished lactic acid production have been found greatly growth delayed and highly infiltrated by ILC2s accompanied by eosinophils following treatment with IL-33. We have therefore identified lactic acid production by melanoma cells as a plausible escape mechanism to evade destruction mediated by ILC2s (60).

Additional studies are necessary to understand the mechanisms involved in the shift of ILC2s from immunosurveillance to immunosuppression associated with the promotion of tumor growth and progression (20, 63).

### ILC3s and LTi Cells

ILC3s have also been accredited a role in melanoma immunosurveillance. For example, a treatment with cyclophosphamide together with an antibody targeting a native melanoma differentiation antigen, tyrosinase-related protein 1 (aTRP1), has been found to inhibit the growth of B16F10 melanoma tumors. It has also been demonstrated that the tumor-suppressing activity of this combined therapy occurs independently of adaptive immunity and NK cells, but is mediated *via* CD90+NK1.1^_^ ILC3s associated with intratumoral macrophage accumulation (64). Additionally, B16F10 melanomas engineered to express IL-12 have been found to initiate local antitumor immunity by stimulating NKp46^+^ ILC3s. Increased accumulation of NKp46^+^ ILC3s has been associated with enhanced infiltration of CD8^+^ and CD4^+^ T cells as well as NK cells coupled with upregulation of adhesion molecules in the vasculature of melanoma tumors. Although T cells have been characterized as the dominant population of infiltrating leukocytes, the growth of melanomas expressing IL-12 has also been inhibited in *Rag1^-/-^* mice, which lack adaptive immunity. Additionally, antibody-mediated depletion experiments ruled out a strong contribution of NK cells in controlling the growth (but not metastatic dissemination) of melanomas expressing IL-12. It has therefore been suggested that NKp46^+^ ILC3s might play a significant antitumor role in the presence of IL-12 (65).

In another study, it has also been revealed that the tissue microenvironment shapes the phenotype of ILC3s. Whereas ILC3s isolated from the spleen have been able to suppress the growth of B16F10 melanoma tumors expressing IL-12, intestinal ILC3s have been found ineffective. Interestingly, transcriptome analysis has revealed mutually exclusive gene expression signatures between the splenic and intestinal ILC3s regarding (but not limited to) leukocyte adhesion and activation. Increased frequencies of leukocytes have been observed in B16F10 melanomas engineered to express IL-12 and co-injected with splenic ILC3s when compared with tumors co-injected with intestinal ILC3s (66).

The involvement of LTi cells in melanoma immunosurveillance remains to be determined. However, it is tempting to hypothesize that LTi cells might contribute to the formation of tertiary lymphoid structures often observed in human melanoma (67).

## Conclusion and Future Prospects

**Figure 1:**
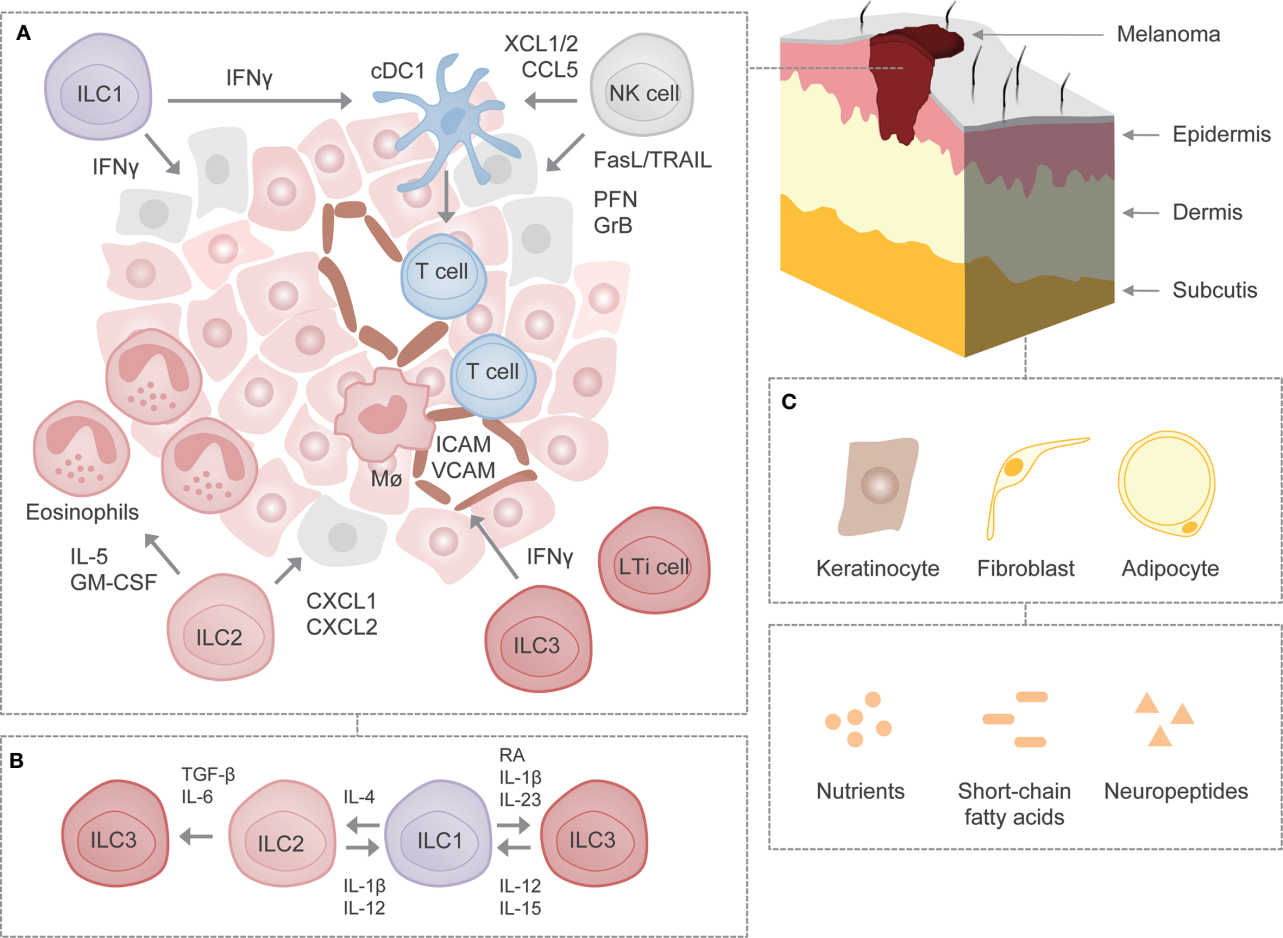
ILCs in melanoma. **(A)** Schematic representation of cancer immunosurveillance by ILCs using mouse melanoma as a model. NK cells may induce apoptosis in melanoma cells through the release of cytotoxic granules containing perforin and granzyme B as well as through the engagement of death receptor-mediated pathways such as TRAIL and FasL. In addition, NK cells may recruit cDC1s to the tumor microenvironment by secreting XCL1/2 and CCL5 and may support their survival and maturation. ILC1s may produce IFN-γ, which exhibits direct antitumor activity or modulates activity of other immune cells. On the other hand, ILC2s may attract and activate eosinophils through the production of IL-5 and GM-CSF. ILC2s may also induce tumor cell-specific apoptosis *via* the release of CXCL1 and CXCL2. ILC3s may stimulate leukocyte recruitment to the tumor microenvironment through IFN-γ-mediated upregulation of adhesion molecules ICAM and VCAM. **(B)** Plastic potential of ILCs. Following stimulation with certain cytokines, growth factors or metabolites, ILCs exhibit potential for plasticity, although it remains to be determined whether ILCs undergo such reversible transdifferentiation in melanoma. **(C)** Interactions with parenchymal cells as well as non-cytokine factors in the skin. It remains to be deciphered whether ILCs interact with certain parenchymal cells as well as non-cytokine factors known to affect the function of ILCs in other settings. FasL, Fas ligand; GrB, granzyme B; ICAM, intercellular adhesion molecule; Mϕ, macrophage; PFN, perforin; RA, retinoic acid; TRAIL, TNF-related apoptosis-inducing ligand; VCAM, vascular cell adhesion molecule.

The involvement of NK cells in antitumor immunity is unquestionable, however, the role and function of other members of the family of ILCs have only recently gained attention. Given the preferential enrichment of ILCs in the skin, a deeper understanding of their contribution to the development and progression of skin malignancies is required. Indeed, a growing body of evidence indicates that ILCs play a critical role in the control of melanoma (**Figure 1**), a type of skin malignancy thought to trigger immunity mediated mainly by adaptive immune responses. However, the extent to which ILCs are engaged in antitumor immunity in general remains vague, as they have been separately associated with both tumor-suppressing and tumor-promoting activities depending on the context of tumor specificity (5, 20).

In order to improve our understanding of ILCs with regard to their role in skin malignancies, it seems imperative to determine how ILCs regulate healthy skin homeostasis. An increasing degree of heterogeneity among ILCs also necessitates their separate assessment in epidermis, dermis and subcutis. Furthermore, studying the mechanisms by which ILCs communicate with other non-hematopoietic parenchymal cells such as keratinocytes, fibroblasts and adipocytes might help to better understand the extent of their involvement in the homeostatic and pathological states (**Figure 1**) (68–70). Recently, it has been revealed that melanoma can arise from melanocyte stem cells found in hair follicles apart from melanocytes found in the bottom layer of epidermis (71). Further investigation should focus on the involvement of ILCs in the early stages of melanoma development. The same holds true for their role and function in the primary site (i.e. skin) as opposed to when confronted by metastases in another tissues.

Studying signals responsible for activation and inhibition of ILCs during malignant development and progression might help to preselect therapeutic targets. Currently, it is unknown how many non-cytokine factors, including nutrients, short-chain fatty acids and neuropeptides, which affect ILC function in other settings, shape ILC responses in melanoma (**Figure 1**). Three-dimensional (3D) spheroid-based *in vitro* models might prove useful during analysis of interactions between ILCs and malignant cells, since culture in 3D has been suggested to affect the expression of molecules involved in melanoma recognition (72–74). Not less important is further investigation of contribution of ILCs to other types of skin cancer, including squamous and basal cell carcinoma, since most of the studies (if not all) have utilized an injectable melanoma model. The methods established so far to identify and characterize ILCs in steady-state (e.g. single cell RNA-sequencing) might pave the way to properly dissect their relevance in cancer. Sophisticated imaging techniques might also allow us to better describe the spatial location of ILCs in the primary and metastatic tumor tissue. The role of specific subsets could be assessed using genetically engineered mice specifically lacking one or the other subset of ILCs. To this end, however, there is a need for identification of unique and highly specific markers. Further compounding the issue is the plastic potential of ILCs, which also merits investigation (**Figure 1**) (5, 6).

Following clarification of the role of ILCs in the skin cancer, translation of the results from mouse models to humans is necessary to fully elucidate the role of ILCs in disease pathogenesis as well as develop potential therapeutic strategies. It remains to be seen whether we can exploit ILCs to maximize its anticancer potential in the clinic.

## Author Contributions

All authors participated in the intellectual conception, critical review and final approval of the manuscript.

## Funding

This work was supported by the FRIPRO Mobility Grant Fellowship from the Research Council of Norway (302241) to MW and by a Grant-in-Aid for Scientific Research (A) (20H00511) from the Japan Society for the Promotion of Science, to SK.

## Acknowledgments

We thank Tetsuro Kobayashi for discussion.

## Conflict of Interest

The authors declare that the research was conducted in the absence of any commercial or financial relationships that could be construed as a potential conflict of interest.

## Publisher’s Note

All claims expressed in this article are solely those of the authors and do not necessarily represent those of their affiliated organizations, or those of the publisher, the editors and the reviewers. Any product that may be evaluated in this article, or claim that may be made by its manufacturer, is not guaranteed or endorsed by the publisher.

## References

[1] Vivier E Artis D Colonna M Diefenbach A Di Santo JP Eberl G. Innate Lymphoid Cells: 10 Years on. Cell (2018) 174(5):1054–66. doi: 10.1016/j.cell.2018.07.017 30142344

[2] Kiniwa T Moro K. Localization and Site-Specific Cell-Cell Interactions of Group 2 Innate Lymphoid Cells. Int Immunol (2021) 33(5):251–9. doi: 10.1093/intimm/dxab001 PMC8060991 33403383

[3] Moro K Yamada T Tanabe M Takeuchi T Ikawa T Kawamoto H. Innate Production of T(H)2 Cytokines by Adipose Tissue-Associated C-Kit(+)Sca-1(+) Lymphoid Cells. Nature (2010) 463(7280):540–4. doi: 10.1038/nature08636 20023630

[4] Bal SM Golebski K Spits H. Plasticity of Innate Lymphoid Cell Subsets. Nat Rev Immunol (2020) 20(9):552–65. doi: 10.1038/s41577-020-0282-9 32107466

[5] Wagner M Moro K Koyasu S. Plastic Heterogeneity of Innate Lymphoid Cells in Cancer. Trends Cancer (2017) 3(5):326–35. doi: 10.1016/j.trecan.2017.03.008 28718410

[6] Bald T Wagner M Gao YL Koyasu S Smyth MJ. Hide and Seek: Plasticity of Innate Lymphoid Cells in Cancer. Semin Immunol (2019) 41:101273. doi: 10.1016/j.smim.2019.04.001 30979591

[7] Ohne Y Silver JS Thompson-Snipes L Collet MA Blanck JP Cantarel BL. IL-1 is a Critical Regulator of Group 2 Innate Lymphoid Cell Function and Plasticity. Nat Immunol (2016) 17(6):646–55. doi: 10.1038/ni.3447 27111142

[8] Bal SM Bernink JH Nagasawa M Groot J Shikhagaie MM Golebski K. IL-1beta, IL-4 and IL-12 Control the Fate of Group 2 Innate Lymphoid Cells in Human Airway Inflammation in the Lungs. Nat Immunol (2016) 17(6):636–45. doi: 10.1038/ni.3444 27111145

[9] Bernink JH Ohne Y Teunissen MBM Wang JY Wu JC Krabbendam L. C-Kit-Positive ILC2s Exhibit an ILC3-Like Signature That may Contribute to IL-17-Mediated Pathologies. Nat Immunol (2019) 20(8):992–1003. doi: 10.1038/s41590-019-0423-0 31263279

[10] Mazzurana L Czarnewski P Jonsson V Wigge L Ringner M Williams TC. Tissue-Specific Transcriptional Imprinting and Heterogeneity in Human Innate Lymphoid Cells Revealed by Full-Length Single-Cell RNA-Sequencing. Cell Res (2021) 31(5):554–68. doi: 10.1038/s41422-020-00445-x PMC8089104 33420427

[11] Meininger I Carrasco A Rao A Soini T Kokkinou E Mjosberg J. Tissue-Specific Features of Innate Lymphoid Cells. Trends Immunol (2020) 41(10):902–17. doi: 10.1016/j.it.2020.08.009 32917510

[12] Kabata H Moro K Koyasu S. The Group 2 Innate Lymphoid Cell (ILC2) Regulatory Network and its Underlying Mechanisms. Immunol Rev (2018) 286(1):37–52. doi: 10.1111/imr.12706 30294963

[13] Klose CSN Artis D. Innate Lymphoid Cells Control Signaling Circuits to Regulate Tissue-Specific Immunity. Cell Res (2020) 30(6):475–91. doi: 10.1038/s41422-020-0323-8 PMC7264134 32376911

[14] Fuchs A Vermi W Lee JS Lonardi S Gilfillan S Newberry RD. Intraepithelial Type 1 Innate Lymphoid Cells are a Unique Subset of IL-12- and IL-15-Responsive IFN-Gamma-Producing Cells. Immunity (2013) 38(4):769–81. doi: 10.1016/j.immuni.2013.02.010 PMC3634355 23453631

[15] Nagasawa M Spits H Ros XR. Innate Lymphoid Cells (ILCs): Cytokine Hubs Regulating Immunity and Tissue Homeostasis. Cold Spring Harb Perspect Biol (2018) 10(12):a030304. doi: 10.1101/cshperspect.a030304 29229782 PMC6280706

[16] Price AE Liang HE Sullivan BM Reinhardt RL Eisley CJ Erle DJ. Systemically Dispersed Innate IL-13-Expressing Cells in Type 2 Immunity. Proc Natl Acad Sci USA (2010) 107(25):11489–94. doi: 10.1073/pnas.1003988107 PMC2895098 20534524

[17] Teng MW Bowman EP McElwee JJ Smyth MJ Casanova JL Cooper AM. IL-12 and IL-23 Cytokines: From Discovery to Targeted Therapies for Immune-Mediated Inflammatory Diseases. Nat Med (2015) 21(7):719–29. doi: 10.1038/nm.3895 26121196

[18] Xue LZ Salimi M Panse I Mjosberg JM McKenzie ANJ Spits H. Prostaglandin D-2 Activates Group 2 Innate Lymphoid Cells Through Chemoattractant Receptor-Homologous Molecule Expressed on T(H)2 Cells. J Allergy Clin Immun (2014) 133(4):1184–94. doi: 10.1016/j.jaci.2013.10.056 PMC3979107 24388011

[19] Tarazona R Duran E Solana R. Natural Killer Cell Recognition of Melanoma: New Clues for a More Effective Immunotherapy. Front Immunol (2015) 6:649. doi: 10.3389/fimmu.2015.00649 26779186 PMC4703774

[20] Wagner M Koyasu S. Cancer Immunoediting by Innate Lymphoid Cells. Trends Immunol (2019) 40(5):415–30. doi: 10.1016/j.it.2019.03.004 30992189

[21] Pietra G Vitale M Moretta L Mingari MC. How Melanoma Cells Inactivate NK Cells. Oncoimmunology (2012) 1(6):974–5. doi: 10.4161/onci.20405 PMC3489764 23162776

[22] Balsamo M Pietra G Vermi W Moretta L Mingari MC Vitale M. Melanoma Immunoediting by NK Cells. Oncoimmunology (2012) 1(9):1607–9. doi: 10.4161/onci.21456 PMC3525618 23264909

[23] Chiossone L Dumas PY Vienne M Vivier E. Natural Killer Cells and Other Innate Lymphoid Cells in Cancer. Nat Rev Immunol (2018) 18(11):671–88. doi: 10.1038/s41577-018-0061-z 30209347

[24] Lopez-Soto A Gonzalez S Smyth MJ Galluzzi L. Control of Metastasis by NK Cells. Cancer Cell (2017) 32(2):135–54. doi: 10.1016/j.ccell.2017.06.009 28810142

[25] Kabashima K Honda T Ginhoux F Egawa G. The Immunological Anatomy of the Skin. Nat Rev Immunol (2019) 19(1):19–30. doi: 10.1038/s41577-018-0084-5 30429578

[26] Slominski AT Brozyna AA Zmijewski MA Jozwicki W Jetten AM Mason RS. Vitamin D Signaling and Melanoma: Role of Vitamin D and its Receptors in Melanoma Progression and Management. Lab Invest (2017) 97(6):706–24. doi: 10.1038/labinvest.2017.3 PMC5446295 28218743

[27] Gudjonsson JE Johnston A Dyson M Valdimarsson H Elder JT. Mouse Models of Psoriasis. J Invest Dermatol (2007) 127(6):1292–308. doi: 10.1038/sj.jid.5700807 17429444

[28] Bruggen MC Bauer WM Reininger B Clim E Captarencu C Steiner GE. In Situ Mapping of Innate Lymphoid Cells in Human Skin: Evidence for Remarkable Differences Between Normal and Inflamed Skin. J Invest Dermatol (2016) 136(12):2396–405. doi: 10.1016/j.jid.2016.07.017 27456756

[29] Kim BS Siracusa MC Saenz SA Noti M Monticelli LA Sonnenberg GF. TSLP Elicits IL-33-Independent Innate Lymphoid Cell Responses to Promote Skin Inflammation. Sci Transl Med (2013) 5(170):170ra16. doi: 10.1126/scitranslmed.3005374 PMC3637661 23363980

[30] Roediger B Kyle R Yip KH Sumaria N Guy TV Kim BS. Cutaneous Immunosurveillance and Regulation of Inflammation by Group 2 Innate Lymphoid Cells. Nat Immunol (2013) 14(6):564–73. doi: 10.1038/ni.2584 PMC4282745 23603794

[31] Kobayashi T Voisin B Kim DY Kennedy EA Jo JH Shih HY. Homeostatic Control of Sebaceous Glands by Innate Lymphoid Cells Regulates Commensal Bacteria Equilibrium. Cell (2019) 176(5):982–97. doi: 10.1016/j.cell.2018.12.031 PMC6532063 30712873

[32] Nagao K Kobayashi T Moro K Ohyama M Adachi T Kitashima DY. Stress-Induced Production of Chemokines by Hair Follicles Regulates the Trafficking of Dendritic Cells in Skin. Nat Immunol (2012) 13(8):744–52. doi: 10.1038/ni.2353 PMC4115277 22729248

[33] Kobayashi T Naik S Nagao K. Choreographing Immunity in the Skin Epithelial Barrier. Immunity (2019) 50(3):552–65. doi: 10.1016/j.immuni.2019.02.023 PMC6455972 30893586

[34] Yang J Hu SM Zhao LM Kaplan DH Perdew GH Xiong N. Selective Programming of CCR10(+) Innate Lymphoid Cells in Skin-Draining Lymph Nodes for Cutaneous Homeostatic Regulation. Nat Immunol (2016) 17(1):48–56. doi: 10.1038/ni.3312 26523865 PMC4838393

[35] Oyesola OO Shanahan MT Kanke M Mooney BM Webb LM Smita S. PGD2 and CRTH2 Counteract Type 2 Cytokine-Elicited Intestinal Epithelial Responses During Helminth Infection. J Exp Med (2021) 218(9):e20202178. doi: 10.1084/jem.20202178 34283207 PMC8294949

[36] DelGiorno KE Chung CY Vavinskaya V Maurer HC Novak SW Lytle NK. Tuft Cells Inhibit Pancreatic Tumorigenesis in Mice by Producing Prostaglandin D2. Gastroenterology (2020) 159(5):1866–81.e8. doi: 10.1053/j.gastro.2020.07.037 32717220 PMC7680354

[37] Maric J Ravindran A Mazzurana L Van Acker A Rao A Kokkinou E. Cytokine-Induced Endogenous Production of Prostaglandin D2 is Essential for Human Group 2 Innate Lymphoid Cell Activation. J Allergy Clin Immunol (2019) 143(6):2202–14.e5. doi: 10.1016/j.jaci.2018.10.069 30578872

[38] Hepworth MR Monticelli LA Fung TC Ziegler CGK Grunberg S Sinha R. Innate Lymphoid Cells Regulate CD4(+) T-Cell Responses to Intestinal Commensal Bacteria. Nature (2013) 498(7452):113–7. doi: 10.1038/nature12240 PMC3699860 23698371

[39] Teunissen MBM Munneke JM Bernink JH Spuls PI Res PCM Te Velde A. Composition of Innate Lymphoid Cell Subsets in the Human Skin: Enrichment of NCR(+) ILC3 in Lesional Skin and Blood of Psoriasis Patients. J Invest Dermatol (2014) 134(9):2351–60. doi: 10.1038/jid.2014.146 24658504

[40] Villanova F Flutter B Tosi I Grys K Sreeneebus H Perera GK. Characterization of Innate Lymphoid Cells in Human Skin and Blood Demonstrates Increase of NKp44+ ILC3 in Psoriasis. J Invest Dermatol (2014) 134(4):984–91. doi: 10.1038/jid.2013.477 PMC3961476 24352038

[41] Bielecki P Riesenfeld SJ Hutter JC Torlai Triglia E Kowalczyk MS Ricardo-Gonzalez RR. Skin-Resident Innate Lymphoid Cells Converge on a Pathogenic Effector State. Nature (2021) 592(7852):128–32. doi: 10.1038/s41586-021-03188 PMC8336632 33536623

[42] Salimi M Barlow JL Saunders SP Xue LZ Gutowska-Owsiak D Wang XW. A Role for IL-25 and IL-33-Driven Type-2 Innate Lymphoid Cells in Atopic Dermatitis. J Exp Med (2013) 210(13):2939–50. doi: 10.1084/jem.20130351 PMC3865470 24323357

[43] Rak GD Osborne LC Siracusa MC Kim BS Wang K Bayat A. IL-33-Dependent Group 2 Innate Lymphoid Cells Promote Cutaneous Wound Healing. J Invest Dermatol (2016) 136(2):487–96. doi: 10.1038/JID.2015.406 PMC4731037 26802241

[44] MacLeod AS Hemmers S Garijo O Chabod M Mowen K Witherden DA. Dendritic Epidermal T Cells Regulate Skin Antimicrobial Barrier Function. J Clin Invest (2013) 123(10):4364–74. doi: 10.1172/JCI70064 PMC3784546 24051381

[45] Li Z Hodgkinson T Gothard EJ Boroumand S Lamb R Cummins I. Epidermal Notch1 Recruits ROR Gamma(+) Group 3 Innate Lymphoid Cells to Orchestrate Normal Skin Repair. Nat Commun (2016) 7:11394. doi: 10.1038/ncomms11394 27099134 PMC4844683

[46] Dvorak HF. Tumors: Wounds That do Not Heal. Similarities Between Tumor Stroma Generation and Wound Healing. N Engl J Med (1986) 315(26):1650–9. doi: 10.1056/NEJM198612253152606 3537791

[47] Boudreau JE Hsu KC. Natural Killer Cell Education and the Response to Infection and Cancer Therapy: Stay Tuned. Trends Immunol (2018) 39(3):222–39. doi: 10.1016/j.it.2017.12.001 PMC6013060 29397297

[48] Lakshmikanth T Burke S Ali TH Kimpfler S Ursini F Ruggeri L. NCRs and DNAM-1 Mediate NK Cell Recognition and Lysis of Human and Mouse Melanoma Cell Lines *In Vitro* and *In Vivo* . J Clin Invest (2009) 119(5):1251–63. doi: 10.1172/JCI36022 PMC2673866 19349689

[49] de Jonge K Ebering A Nassiri S Maby-El Hajjami H Ouertatani-Sakouhi H Baumgaertner P. Circulating CD56(bright) NK Cells Inversely Correlate With Survival of Melanoma Patients. Sci Rep (2019) 9(1):4487. doi: 10.1038/s41598-019-40933-8 30872676 PMC6418246

[50] Barry KC Hsu J Broz ML Cueto FJ Binnewies M Combes AJ. A Natural Killer-Dendritic Cell Axis Defines Checkpoint Therapy-Responsive Tumor Microenvironments. Nat Med (2018) 24(8):1178–91. doi: 10.1038/s41591-018-0085-8 PMC6475503 29942093

[51] Bottcher JP Bonavita E Chakravarty P Blees H Cabeza-Cabrerizo M Sammicheli S. NK Cells Stimulate Recruitment of Cdc1 Into the Tumor Microenvironment Promoting Cancer Immune Control. Cell (2018) 172(5):1022–37.e14. doi: 10.1016/j.cell.2018.01.004 29429633 PMC5847168

[52] Ercolano G Garcia-Garijo A Salome B Gomez-Cadena A Vanoni G Mastelic-Gavillet B. Immunosuppressive Mediators Impair Proinflammatory Innate Lymphoid Cell Function in Human Malignant Melanoma. Cancer Immunol Res (2020) 8(4):556–64. doi: 10.1158/2326-6066.CIR-19-0504 32019778

[53] Benci JL Johnson LR Choa R Xu Y Qiu J Zhou Z. Opposing Functions of Interferon Coordinate Adaptive and Innate Immune Responses to Cancer Immune Checkpoint Blockade. Cell (2019) 178(4):933–48.e14. doi: 10.1016/j.cell.2019.07.019 31398344 PMC6830508

[54] Kim J Kim W Moon UJ Kim HJ Choi HJ Sin JI. Intratumorally Establishing Type 2 Innate Lymphoid Cells Blocks Tumor Growth. J Immunol (2016) 196(5):2410–23. doi: 10.4049/jimmunol.1501730 26829987

[55] Rosenberg HF Dyer KD Foster PS. Eosinophils: Changing Perspectives in Health and Disease. Nat Rev Immunol (2013) 13(1):9–22. doi: 10.1038/nri3341 23154224 PMC4357492

[56] Ikutani M Yanagibashi T Ogasawara M Tsuneyama K Yamamoto S Hattori Y. Identification of Innate IL-5-Producing Cells and Their Role in Lung Eosinophil Regulation and Antitumor Immunity. J Immunol (2012) 188(2):703–13. doi: 10.4049/jimmunol.1101270 22174445

[57] Jacquelot N Seillet C Wang M Pizzolla A Liao Y Hediyeh-Zadeh S. Blockade of the Co-Inhibitory Molecule PD-1 Unleashes ILC2-Dependent Antitumor Immunity in Melanoma. Nat Immunol (2021) 22(7):851–64. doi: 10.1038/s41590-021-00943-z PMC7611091 34099918

[58] Lucarini V Ziccheddu G Macchia I La Sorsa V Peschiaroli F Buccione C. IL-33 Restricts Tumor Growth and Inhibits Pulmonary Metastasis in Melanoma-Bearing Mice Through Eosinophils. Oncoimmunology (2017) 6(6):e1317420. doi: 10.1080/2162402X.2017.1317420 28680750 PMC5486175

[59] Mattes J Hulett M Xie W Hogan S Rothenberg ME Foster P. Immunotherapy of Cytotoxic T Cell-Resistant Tumors by T Helper 2 Cells: An Eotaxin and STAT6-Dependent Process. J Exp Med (2003) 197(3):387–93. doi: 10.1084/jem.20021683 PMC2193835 12566422

[60] Wagner M Ealey KN Tetsu H Kiniwa T Motomura Y Moro K. Tumor-Derived Lactic Acid Contributes to the Paucity of Intratumoral ILC2s. Cell Rep (2020) 30(8):2743–57.e5. doi: 10.1016/j.celrep.2020.01.103 32101749

[61] Moreira A Leisgang W Schuler G Heinzerling L. Eosinophilic Count as a Biomarker for Prognosis of Melanoma Patients and its Importance in the Response to Immunotherapy. Immunotherapy (2017) 9(2):115–21. doi: 10.2217/imt-2016-0138 28128709

[62] Grisaru-Tal S Itan M Klion AD Munitz A. A New Dawn for Eosinophils in the Tumour Microenvironment. Nat Rev Cancer (2020) 20(10):594–607. doi: 10.1038/s41568-020-0283-9 32678342

[63] Long A Dominguez D Qin L Chen S Fan J Zhang M. Type 2 Innate Lymphoid Cells Impede IL-33-Mediated Tumor Suppression. J Immunol (2018) 201(11):3456–64. doi: 10.4049/jimmunol.1800173 PMC6264920 30373846

[64] Moskalenko M Pan M Fu Y de Moll EH Hashimoto D Mortha A. Requirement for Innate Immunity and CD90(+) NK1.1(-) Lymphocytes to Treat Established Melanoma With Chemo-Immunotherapy. Cancer Immunol Res (2015) 3(3):296–304. doi: 10.1158/2326-6066.CIR-14-0120 25600438 PMC4690202

[65] Eisenring M vom Berg J Kristiansen G Saller E Becher B. IL-12 Initiates Tumor Rejection *via* Lymphoid Tissue-Inducer Cells Bearing the Natural Cytotoxicity Receptor Nkp46. Nat Immunol (2010) 11(11):1030–8. doi: 10.1038/ni.1947 20935648

[66] Nussbaum K Burkhard SH Ohs I Mair F Klose CSN Arnold SJ. Tissue Microenvironment Dictates the Fate and Tumor-Suppressive Function of Type 3 ILCs. J Exp Med (2017) 214(8):2331–47. doi: 10.1084/jem.20162031 PMC5551572 28698286

[67] Werner F Wagner C Simon M Glatz K Mertz KD Laubli H. A Standardized Analysis of Tertiary Lymphoid Structures in Human Melanoma: Disease Progression- and Tumor Site-Associated Changes With Germinal Center Alteration. Front Immunol (2021) 12. doi: 10.3389/fimmu.2021.675146 PMC8264652 34248957

[68] Wagner M Bjerkvig R Wiig H Melero-Martin JM Lin RZ Klagsbrun M. Inflamed Tumor-Associated Adipose Tissue is a Depot for Macrophages That Stimulate Tumor Growth and Angiogenesis. Angiogenesis (2012) 15(3):481–95. doi: 10.1007/s10456-012-9276-y PMC3619408 22614697

[69] Wagner M Bjerkvig R Wiig H Dudley AC. Loss of Adipocyte Specification and Necrosis Augment Tumor-Associated Inflammation. Adipocyte (2013) 2(3):176–83. doi: 10.4161/adip.24472 PMC3756107 23991365

[70] Wagner M Dudley AC. A Three-Party Alliance in Solid Tumors: Adipocytes, Macrophages and Vascular Endothelial Cells. Adipocyte (2013) 2(2):67–73. doi: 10.4161/adip.23016 23805401 PMC3661111

[71] Sun Q Lee W Mohri Y Takeo M Lim CH Xu XW. A Novel Mouse Model Demonstrates That Oncogenic Melanocyte Stem Cells Engender Melanoma Resembling Human Disease. Nat Commun (2019) 10(1):5023. doi: 10.1038/s41467-019-12733-1 PMC6828673 31685822

[72] Wagner M Koyasu S. A 3d Skin Melanoma Spheroid-Based Model to Assess Tumor-Immune Cell Interactions. Bio Protoc (2020) 10(23):e3839. doi: 10.21769/BioProtoc.3839 PMC7842405 33659488

[73] Feder-Mengus C Ghosh S Weber WP Wyler S Zajac P Terracciano L. Multiple Mechanisms Underlie Defective Recognition of Melanoma Cells Cultured in Three-Dimensional Architectures by Antigen-Specific Cytotoxic T Lymphocytes. Br J Cancer (2007) 96(7):1072–82. doi: 10.1038/sj.bjc.6603664 PMC2360115 17342088

[74] Ghosh S Rosenthal R Zajac P Weber WP Oertli D Heberer M. Culture of Melanoma Cells in 3-Dimensional Architectures Results in Impaired Immunorecognition by Cytotoxic T Lymphocytes Specific for Melan-A/MART-1 Tumor-Associated Antigen. Ann Surg (2005) 242(6):851–8. doi: 10.1097/01.sla.0000189571.84213.b0 PMC1409875 16327495

